# Symptoms presented during emergency telephone calls for patients with spontaneous subarachnoid haemorrhage

**DOI:** 10.1186/s13049-021-00934-x

**Published:** 2021-08-16

**Authors:** Asger Sonne, Sarita Egholm, Laurits Elgaard, Niklas Breindahl, Alice Herrlin Jensen, Vagn Eskesen, Freddy Lippert, Frans Boch Waldorff, Nicolai Lohse, Lars Simon Rasmussen

**Affiliations:** 1 Department of Anaesthesia, section 6011, Center of Head and Orthopaedics, Rigshospitalet, Inge Lehmanns Vej 6, 2100 Copenhagen, Denmark; 2 Department of Neurosurgery, The Neuroscience Centre, Rigshospitalet, University of Copenhagen, Copenhagen, Denmark; 3 Copenhagen Emergency Medical Services, Copenhagen, Denmark; 4 Research Unit of General Practice, Department of Public Health, University of Southern Denmark, Odense, Denmark; 5 The Research Unit for General Practice and Section of General Practice, Department of Public Health, University of Copenhagen, Copenhagen, Denmark; 6 Department of Emergency Medicine, Copenhagen University Hospital – Nordsjællands Hospital, Hillerød, Denmark; 7 Department of Clinical Medicine, University of Copenhagen, Copenhagen, Denmark

**Keywords:** Spontaneous subarachnoid haemorrhage, Emergency medical service, Emergency medical dispatch, Symptoms, Headache, Telephone, Triage, Visitation

## Abstract

**Background:**

A spontaneous subarachnoid haemorrhage (SAH) is one of the most critical neurological emergencies a dispatcher can face in an emergency telephone call. No study has yet investigated which symptoms are presented in emergency telephone calls for these patients. We aimed to identify symptoms indicative of SAH and to determine the sensitivity of these and their association (odds ratio, OR) with SAH.

**Methods:**

This was a nested case–control study based on all telephone calls to the medical dispatch center of Copenhagen Emergency Medical Services in a 4-year time period. Patients with SAH were identified in the Danish National Patient Register; diagnoses were verified by medical record review and their emergency telephone call audio files were extracted. Audio files were replayed, and symptoms extracted in a standardized manner. Audio files of a control group were replayed and assessed as well.

**Results:**

We included 224 SAH patients and 609 controls. Cardiac arrest and persisting unconsciousness were reported in 5.8% and 14.7% of SAH patients, respectively. The highest sensitivity was found for headache (58.9%), nausea/vomiting (46.9%) and neck pain (32.6%). Among conscious SAH patients these symptoms were found to have the strongest association with SAH (OR 27.0, 8.41 and 34.0, respectively). Inability to stand up, speech difficulty, or sweating were reported in 24.6%, 24.2%, and 22.8%. The most frequent combination of symptoms was headache and nausea/vomiting, which was reported in 41.6% of SAH patients. More than 90% of headaches were severe, but headache was not reported in 29.7% of conscious SAH patients. In these, syncope was described by 49.1% and nausea/vomiting by 37.7%.

**Conclusion:**

Headache, nausea/vomiting, and neck pain had the highest sensitivity and strongest association with SAH in emergency telephone calls. Unspecific symptoms such as inability to stand up, speech difficulty or sweating were reported in 1 out of 5 calls. Interestingly, 1 in 3 conscious SAH patients did not report headache.

*Trial registration* NCT03980613 (www.clinicaltrials.gov).

## Introduction

In many countries, citizens can call an emergency telephone number if they are in urgent need of the emergency medical service (EMS). The call is usually answered by an emergency medical dispatcher (EMD) in an emergency medical dispatch center (EMDC). The EMD must assess the level of emergency, dispatch the appropriate prehospital resources and provide advice to the caller until the EMS arrives. Compared to face-to-face visitation, this telephone visitation is challenging due to the lack of nonverbal and visual cues that are normally a part of clinical decision making [1]. One of the most time-critical neurological emergencies EMDs can face is a spontaneous subarachnoid haemorrhage (SAH). These patients may present with a variety of symptoms. In the most severe cases, patients lose consciousness or go into cardiac arrest [2]. Others are conscious and describe the worst headache of their life, while others again report less severe and unspecific symptoms [3]. The wide spectrum of presentations makes SAH a challenging condition to recognize, especially among conscious patients with less severe symptoms which accounts for up to half of all SAH patients [4, 5]. The uncharacteristic and diverse symptoms may result in initial under-triage and subsequent treatment delays even when patients are seen face-to-face by medical professionals in emergency departments [6]. In addition, SAH is a rare event with an incidence rate of 5.5 per 100,000 person-years [7] and consequently EMDs will infrequently encounter these patients. Our knowledge of SAH symptoms is predominantly based on retrospective studies [8, 9] and patients’ presentations in emergency department settings [10]. We have only limited knowledge about SAH symptoms in the acute phase as experienced during an emergency telephone call. Consequently, the primary aim of this study was to identify symptoms and combinations of symptoms, indicative of SAH during emergency telephone calls. Second, we aimed to determine the sensitivity of these symptoms and their association with SAH. Finally, we aimed to identify factors in the telephone visitation that may influence the level of urgency of the activated prehospital response.

## Methods

This was a nested case–control study based on all telephone calls to the EMDC in Copenhagen between 2015 and 2018. Data extraction was performed between 26 August 2019 and 9 January 2021.

### Setting

If a citizen calls the emergency number ‘1-1-2’ in Denmark with a medical emergency, the call is answered by an EMD at the regional EMDC. The Copenhagen EMDC handles approximately 105,000 emergency calls and more than 900,000 calls to the non-urgent medical help line ‘1813’ yearly [11, 12]. Both numbers are available 24/7. If the call-taker perceives a call to the non-urgent medical help line to be an emergency situation they can convert the call to a high priority emergency call. In addition, ambulance requests from general practitioners, other health care providers or police are also handled by the Copenhagen EMDC, but these are handled directly without further assessment by an EMD. EMDs are registered nurses or paramedics. They receive six weeks of training in telephone triage and the use of the electronic decision support system Danish Index for Emergency Care [13]. The index is a criteria-based system that gives advice to appropriate prehospital response based on the patients’ primary complaint or the EMDs clinical suspicion. The index is divided into 37 overall chapters, each with numerous symptoms-specific subcategories. Audio files of the telephone calls are automatically logged.

### Cases and controls

Cases were defined as patients identified in the Danish National Patient Register and with an emergency telephone call to the Copenhagen EMDC immediately before their admission. They were aged 18 years or more at the time of admission, were admitted to one of the nine hospitals in the Capital Region of Denmark between 1 January 2015 and 31 December 2018 and discharged with a diagnosis of non-traumatic SAH [International Classification of Diseases version 10 (ICD-10) codes I60.0-I60.9]. The list was cross-referenced with SAH patients seen at the Department of Neurosurgery and Neurointensive Care at Copenhagen University Hospital Rigshospitalet, the only center for centralized and highly specialised treatment and critical care within the region. Cross-referencing was done to check where patients had been admitted and thus from where their medical records could be accessed. We included both patients who had been admitted to highly specialised care and those who remained at referring hospitals. Two independent reviewers (LE and SE) screened every medical record following a structured case report form to verify diagnoses. Their findings were entered into the data collection software Research Electronic Datacapture (RedCap 10.3.3, Vanderbilt University) and compared. In cases of disagreement a third party (AS) was consulted and in neurosurgical/neurological matters a consultant neurosurgeon (VE) performed an additional medical record review. A verified diagnosis required a computed tomography scan or xantochromia on spinal fluid analysis as the basis for diagnosing SAH. We excluded patients with SAH earlier in their life, those transferred from other geographical regions, traumatic SAH, reversible cerebral vasoconstriction syndrome, tumour haemorrhages, and spinal SAHs. If patients had made several calls, only the call leading to admission was included. Controls were selected at random among patients without SAH who had called the EMDC within the study period. They were at least 18 years of age and had been assigned one of the following overall Danish Index for Emergency Care chapters: persisting unconsciousness, unclear problem, headache, seizure, reduced consciousness/ paralysis. These overall chapters were chosen as they were believed to be the most commonly assigned to patients with SAH calling the EMDC. No matching was performed as to best mimic the general cohort of callers within these overall chapters. The reporting of the association between symptoms and SAH was done separately for conscious patients (including those with brief syncope) and patients with persisting unconsciousness.

### Audio file data extraction

Audio files of telephone calls were extracted from the EMDC for both cases and controls. Two investigators (AJ and NB) listened to half of the audio files each, blinded to whether calls were from cases or controls. Blinding was done by renaming audio files. The two investigators extracted data from the audio files using a standardized RedCap data collection form. Variables for the data collection form were SAH symptoms described in the literature [2, 3, 8–10, 14] and symptoms identified by analysing twenty-five randomly selected emergency calls from SAH patients. It was also recorded who the EMD was talking to (i.e., the patient or a bystander), if there were major communication issues, and the duration of symptoms. If headache was described, also the time to peak intensity, the location and the severity was recorded if they were available. After designing the data collection form, interrater agreement of all variables was assessed. Cohen´s kappa (κ) was used for categorical data and weighted κ for ordinal data. The data collection form was optimized multiple times and interrater agreement re-evaluated. Rarely does an emergency telephone call include information on the absence of symptoms as focus is often on the symptoms present. Therefore, we registered only reported symptoms and not the absence of symptoms.

### Survival and comorbidity

Thirty-day survival was extracted from the The Danish Civil Registration System [15] which is updated daily and has near complete follow-up. As perimesencephalic haemorrhages and haemorrhages with no identified source are generally considered non-lethal, 30-day survival was reported separately for these. Charlson Comorbidity Index scores were computed from data extracted from the Danish National Patient Register during the last 10 years prior to the SAH. We used the ICD-10 translation of Deyo’s coding algorithm developed by Sundararajan [16].

### Statistics

We assumed that symptoms of interest would occur in 25% of patients with SAH and in five percent of controls. To detect a difference of this magnitude with 80% power at the five percent significance level, we decided to include 195 cases and 390 controls (ratio 1:2). Comparison of proportions was done by the χ^2^-test and continuous data were compared using the Mann–Whitney U-test. *P* < 0.05 was considered significant. Sensitivities with 95% confidence intervals (CI) of symptoms were reported for all SAH patients. Crude odds ratios (OR) with 95% CI were reported separately for conscious patients (including brief syncope) and patients in cardiac arrest/persistently unconscious. The latter was done to reflect the two very different situations of triaging conscious patients versus unconscious patients. Interrater agreements were reported as κ-values. κ > 0.5 was considered acceptable as this indicated a fair to excellent agreement [17]. Predictors for the level of activated prehospital response dispatched to conscious patients were analysed in a stepwise selection logistic regression model. Independent variables were symptoms; sex; age (in decades); communication problems between caller and dispatcher; time period from symptom onset to emergency call, and whom the dispatcher was talking to. The outcome variable of interest was an ambulance response with lights and sirens being dispatched. Results were reported as adjusted odds ratios with 95% confidence intervals.

Statistical analyses were performed in SAS Enterprise Guide 7.1.

### Ethics

The Committees on Health Research Ethics for the Capital Region of Denmark waived the need for approval. Authorization to data access was granted by the Danish Patient Safety Authority and the Danish Data Protection Agency.

### Funding

Funding was received from the Danish non-profit organization TrygFonden.

## Results

**Fig. 1:**
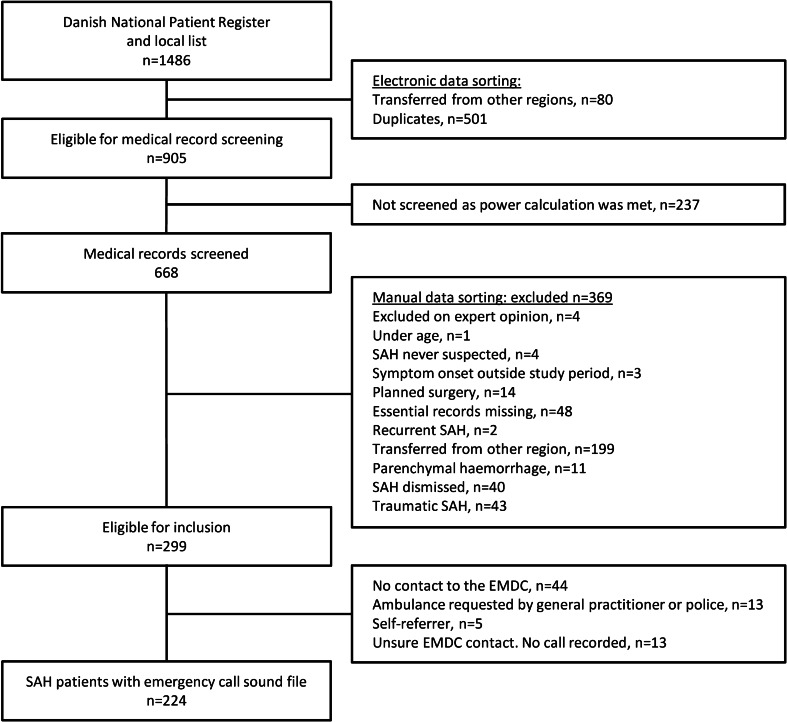
Inclusion flow chart for study of patients with subarachnoid haemorrhage (SAH). *n* number, *EMDC* Emergency Medical Coordination Center

We extracted a total of 1429 patients from the Danish National Patient Register with a diagnosis of SAH within the study period. In addition, 57 patients who were not in the register were identified at the Department of Neurosurgery and Neurointensive Care (Fig. 1). By the time 668 patients’ medical records were screened, 299 were eligible for inclusion and of these 224 patients were included. Then, the inclusion stopped, leaving 237 patients’ medical records unscreened. The 668 screened patients and the 237 not screened were comparable with regards to sex (57.3% and 58.6% females respectively, *P* = 0.72) and age (median 59.4 and 61.1 years respectively, *P* = 0.15). The type of haemorrhage for the 299 eligible patients is reported in Table 1.

**Table 1:** Aetiology of the spontaneous subarachnoid haemorrhages in patients calling the Emergency Medical Dispatch Center

Haemorrhage type	n	%	95% CI
Aneurysmal	209	70.4	64.8–75.5
No haemorrhage site identified	51	17.1	12.9–21.8
Perimesencephalic haemorrhage	24	8.0	5.2–11.7
Arterial dissection	7	2.3	0.9–4.8
Arterio-venous malformation	6	2.0	0.7–4.3
Missing	2	0.7	0.1–2.4

n = 299. *n* number, *CI* confidence interval

Their median Charlson Comorbidity Index score was 0 (Inter-quartile range: 0–0). Thirty-day survival was 100% (95% CI 85.75–100) for perimesencephalic haemorrhages, 80.39% (95% CI 66.88–90.18) for haemorrhages with no identified source and 86.16% (95% CI 80.93–90.40) for all others. Controls were identified at the time cases were deemed eligible for inclusion. This amounted to 609 controls. As some cases later turned out not to have a registered contact with the EMDC and other cases were excluded late in the process, there were on average 2.7 controls per case. For demographic descriptions of cases and controls see Table 2.

**Table 2:** Demographic data for cases and controls

	n	Age (median)	Age (IQR)	Females %, (n)
Cases	224	59.7	47.3–68.5	60.7 (136)
Controls	609	67.9	50.8–79.1	49.4 (301)

Cardiac arrest and persisting unconsciousness were reported in 5.8% (95% CI 3.1–9.7) and 14.7% (95% CI 10.4–20.1) of SAH patients, respectively. We found the highest sensitivity for the following symptoms: headache (58.9%, 95% CI 52.2–65.4), nausea/vomiting (46.9%, 95% CI 40.2–53.6) and neck pain (32.6%, 95% CI 26.5–39.2). Other common symptoms in these patients included inability to stand up, speech difficulty, or sweating (Table 3).

**Table 3:** The sensitivity of symptoms reported during emergency calls to an emergency medical dispatch center by patients with spontaneous subarachnoid haemorrhage (n = 224) and controls (n = 609)

Symptom	Cases			Controls		
	n	%	95% CI	n	%	95% CI
Headache	132	58.9	52.2–65.4	41	6.7	4.9–9.0
Nausea/vomiting	105	46.9	40.2–53.6	63	10.3	8.0–13.0
Neck pain	73	32.6	26.5–39.2	10	1.6	0.8–3.0
Unable to stand/walk	55	24.6	19.1–30.7	98	16.1	13.3–19.3
Sweating	51	22.8	17.5–28.8	69	11.3	8.9–14.1
Dizziness	49	21.9	16.6–27.9	113	18.6	15.5–21.9
Speech difficulty	43	19.2	14.3–24.9	91	14.9	12.2–18.0
Syncope	40	17.9	13.1–23.5	154	25.3	21.9–28.9
Dyspnoea	34	15.2	10.8–20.6	88	14.5	11.8–17.5
Persistently unconscious	33	14.7	10.4–20.1	68	11.2	8.8–13.9
Fatigued/tired	25	11.2	7.4–16.0	96	15.8	12.9–18.9
Feverish	14	6.3	3.5–10.3	29	4.8	3.2–6.8
Cardiac arrest	13	5.8	3.1–9.7	30	4.9	3.4–6.9
Fecal incontinence	11	4.9	2.5–8.6	12	1.9	1.0–3.4
Back pain	10	4.5	2.2–8.1	16	2.6	1.5–4.2
Facial paralysis	9	4.0	1.9–7.5	44	7.2	5.3–9.6
Visual disturbance	6	2.7	0.9–5.7	12	1.9	1.0–3.4
Abdominal pain	4	1.8	0.5–4.5	15	2.5	1.4–4.0
Cardiac arrythmia	2	0.9	0.1–3.2	12	1.9	1.0–3.4
Photophobia	1	0.5	0.0–2.5	2	0.3	0.0–1.2
Chest pain	0	0	–	23	3.8	2.4–5.6

*EMDC* Emergency Medical Dispatch Center, *n* number, *CI* confidence interval

Thirty controls had a cardiac arrest, 68 were persistently unconscious and 511 were conscious. Any two-symptoms combination described by more than ten percent of conscious SAH patients (n = 178, 79.4%) is reported in Table 4.

**Table 4:** Symptoms combinations reported by 174 conscious patients with spontaneous subarachnoid haemorrhage

Symptom/symptom [% (n)]	Neck pain	Headache	Nausea/vomiting
Headache	37.6 (67)	–	–
Nausea/vomiting	25.3 (45)	41.6 (74)	–
Sweating	15.7 (28)	23.6 (42)	16.9 (30)
Unable to stand/walk	11.8 (21)	23.0 (41)	16.9 (30)
Speech difficulty	< 10%	14.6 (26)	11.8 (21)
Dizziness	17.9 (32)	21.4 (38)	19.1 (34)
Fatigued/tired	< 10%	10.1 (18)	< 10%
Syncope	< 10%	< 10%	11.2 (20)

Any two-symptom combination reported by at least 10% of patients is reported

The most frequent combination was headache and nausea/vomiting, which was reported in 41.6% of cases, followed by headache combined with neck pain in 37.6%. Of the 178 conscious patients, 53 (29.7%) did not complain of headache. Syncope and nausea/vomiting were the most frequently occurring symptoms among these (49.0% and 37.7%, respectively) (Table 5).

**Table 5:** Symptoms reported by 53 conscious patients with spontaneous subarachnoid haemorrhage but no headache

Symptoms	n	%	95% CI
Syncope	26	49.1	35.1–63.2
Nausea/vomiting	20	37.7	24.8–52.1
Speech difficulty	17	32.1	19.9–46.3
Unable to stand/walk	14	26.4	15.3–40.3
Dizziness	9	16.9	8.1–29.8
Fatigue/tired	7	13.2	5.5–25.3
Dyspnoea	7	13.2	5.5–25.3
Sweating	6	11.3	4.3–23.0
Neck pain	5	9.4	3.1–20.7
Facial paralysis	4	7.6	2.1–18.2
Fecal incontinence	3	5.7	1.2–15.7
Feverish	2	3.8	0.5–12.9
Back pain	2	3.8	0.5–12.9
Visual disturbances	2	3.8	0.5–12.9
Abdominal pain	1	1.9	0.1–10.1

**Fig. 2:**
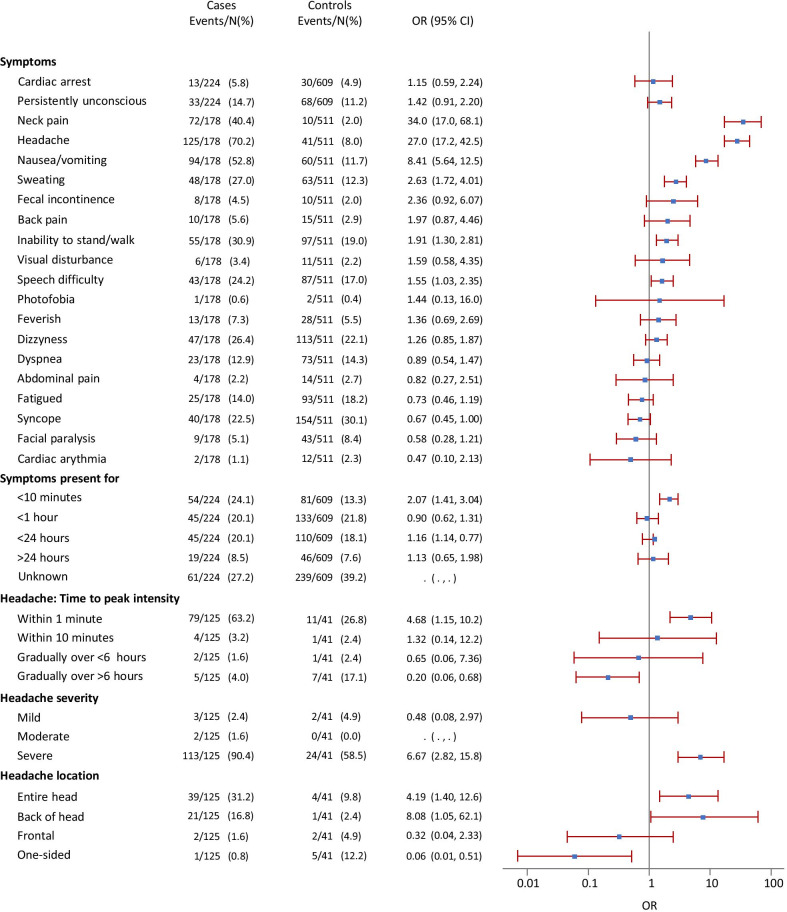
Symptoms reported by patients with spontaneous subarachnoid haemorrhage and controls, in calls to the Emergency Medical Coordination Center. Crude odds ratios are presented with 95% confidence intervals. Two hundred twenty-four cases were included of which 178 were conscious. *N* number, *OR* odds ratio, *95% CI* 95% confidence interval

Among controls, the selected Danish Index for Emergency Care chapters were used in the following proportions of calls: persisting unconsciousness (12.2%), unclear problem (36.8%), headache (1.6%), seizure (8.9%) and reduced consciousness/paralyses (40.6%). An ambulance was dispatched to 93.2% of controls. Odds ratios for the association between SAH and symptoms are reported in Fig. 2. Conscious patients (n = 178, 79.5%) were analysed separately from those who were in cardiac arrest (n = 13, 5.8%) or persistently unconscious for other reasons (n = 33, 14.7%). One hundred seventy-six (78.6%, 95% CI 72.6–83.8) SAH patients received an ambulance with lights and sirens. In conscious patients, only symptom onset within 10 min independently increased the chance of receiving an ambulance with lights and sirens (OR 4.4, 95% CI 1.1–17.1, *P* = 0.0004). On the contrary, the chance was reduced if the caller reported that the patient was conscious but “unable to stand up or walk” (OR 0.2, 95% CI 0.1–0.5, *P* < 0.0001). The same was observed if symptom onset was more than 24 h ago (OR 0.2, 95% CI 0.1–0.7, *P* = 0.0004), if nausea/vomiting (OR 0.4, 95% CI 0.2–0.8, *P* = 0.0095) or back pain (OR 0.1, 95% CI 0.0–0.3, *P* = 0.0001) was reported. Among those who did not receive an ambulance with lights and sirens (n = 48) the most common symptoms were severe headache (60.4%, 95% CI 45.3–74.2, n = 29), nausea/vomiting (62.5%, 95% CI 47.4–76.1, n = 30) and neck pain (35.4%, 95% CI 22.2–50.5, n = 17). Twenty-six percent (n = 58) of SAH patients were initially brought to a hospital with neurosurgical/neurointensive care facilities. Interrater agreement of symptoms reported in emergency calls was good (i.e. κ > 0.75) for most variables regarding symptoms, while it was fair to good (i.e. κ = 0.50–0.75) for variables pertaining to circumstances surrounding the call. Interrater agreement was low for “physical activity level at symptom onset” (κ = 0.37) and “confusion” (κ = 0.47) and they were omitted from the analyses.

## Discussion

In this nested case–control study of 224 SAH patients´ and 609 controls´ emergency telephone calls to the EMDC, we found headache, nausea/vomiting and neck pain to have the highest sensitivities and strongest associations with SAH. A broad variety of symptoms, symptom combinations, and symptom durations were identified. Finally, if symptoms had lasted less than ten minutes there was a greater chance of receiving an ambulance with lights and sirens. Our study has several strengths, the first being that we were able to track patients across numerous registries with minimal loss to follow-up. Also, we studied a well-defined geographical region, with all emergency calls and ambulance requests coming through one EMDC. In addition, great care was taken to verify diagnoses and ensure a uniform data collection. Our study is subject to limitations as well. Patients were identified from two sources that both required the patients to be alive until hospital admission. In a previous study we found that only 3.5% of patients dying from SAH before hospital admission had called the EMDC within the preceding 72 h [18]. We therefore assume that very few SAH patients who may have called the EMDC were missed in the present study. We would have liked to record the haemorrhage severity at the time of the emergency call. This, however, was not possible as the level of detail in emergency calls did not allow a classification according to any recognized SAH severity scale. Having reported the severity upon hospital arrival would not necessarily reflect the severity at the time of the call. Another limitation is the choice of controls. These were randomly picked among patients who were assigned one of five predefined chapters in the decision support system used at the EMDC. This poses a risk of over-representation of the reported symptoms in the control group. Only a small proportion of SAH patients presented with complaints that fell outside of these chapters. The reported prevalence of symptoms may also not be directly extrapolated to that of emergency calls to EMDCs using different interview techniques and different decision support algorithms. Finally, two interesting variables did not fulfil our predefined criterion for interrater agreement level and had to be omitted. The sensitivity of headache was 59%. This is markedly lower than in several other studies, which have reported sensitivities between 74% and 86% [3, 8–10]. These studies were based on emergency department medical records and the difference may thus indicate an under-reporting of headache in emergency calls. The sensitivities of other “classic” SAH symptoms such as nausea/vomiting, neck pain, syncope and persisting unconsciousness are consistent with those reported in studies based on retrospective reports and emergency department observations [3, 8]. Interestingly, frequently occurring symptoms in emergency calls were sudden sweating, inability to stand up or walk among conscious patients, dizziness, speech difficulty and dyspnoea; each of these occurred in 15–25% of conscious patients. Aside from dyspnoea these symptoms were significantly associated with SAH. They often occurred in combination with headache, neck pain or nausea/vomiting. Up to half of all patients with SAH are known to present to health care providers in an intact neurological state [5]. This is a particularly high-risk group of patients, as they are more often misdiagnosed, their treatment delayed and their outcomes worse [14]. We found that 53 out of 178 (29.7%) conscious SAH patients did not complain of headache. It is generally assumed that no more than 10% present without headache at the time of admission [9], and in that respect telephone triage may differ significantly from face-to-face examinations. Patients without headache seem to have higher rates of misdiagnoses, delayed diagnoses, rebleedings, neurological deterioration before admission, lower rates of successful aneurysm repair, increased mortality and poorer neurological outcome in survivors [9, 19]. The larger proportion of patients not reporting headache in our study compared to studies based on emergency department presentations give notion to the thought that some information never came up during the conversation with the EMD. Patients not reporting any headache often presented with brief syncope, nausea/vomiting or sudden fatigue; symptoms normally associated with benign medical conditions. This was also reflected in our analysis of factors affecting ambulance response. Here, the presence of nausea/vomiting, back pain, being conscious but unable to stand up/walk and onset of symptoms more than 24 h ago all reduced the chance of getting an ambulance with lights and sirens. It is not realistic to admit all callers with these symptoms with lights and sirens without an inappropriate level of over-triage. Yet, as 21% did not receive an ambulance with lights and sirens, despite having symptoms similar to those that did receive an urgent ambulance, there is room for improvement. In ischaemic stroke, improved recognition by EMDs has resulted in a shorter ambulance response times, shorter on-scene times, earlier arrival to stroke centers, and faster inhospital responses through pre-arrival notifications. The EMDs are crucial in this process, as any information obtained may be forwarded to the EMS to assist them in choosing the appropriate hospital [20–23]. This is also very likely to be the case with SAH. We found that 75% of SAH patients were admitted by calling the EMDC. There are no comparable studies on SAH, but two Norwegian studies found that only 45–48% of stroke patients were admitted through the EMDC [22, 24]. More severe symptoms are known to be associated with calling the EMDC as the first medical contact [21, 24]. This indicates that patients or bystanders may experience the presentation of SAH as being more severe than stroke in general. We found that 44 patients were admitted without calling the EMDC and no other health care provider requested an ambulance. It would have been interesting to know in detail how and why they were admitted. In our EMDC EMDs are guided by an electronic decision support system using criteria-based dispatch. EMDs decisions are based on a complex interaction between knowledge, clinical experience, and support from the system [22]. One way to improve recognition of SAH patients would be by feedback, focused training and internal audits as suggested by Viereck [11] in a study on cardiac arrest recognition. No SAH specific scoring system have yet been developed to aid EMDs in the early recognition of SAH patients. A support tool like that is particularly necessary to identify conscious, neurologically intact SAH patients calling the EMDC. In patients without headache, neck pain or nausea/vomiting may be present in combination with one or more of the symptoms in Table 3, which may lead to a suspicion of SAH. The best validated emergency department tool to identify SAH patients is the Ottawa SAH rule [5]. It was developed to screen those with a new headache peaking within one hour. Applying the interview part of this tool to the EMDC decision support system, with the addition of questions of nausea/vomiting; sweating; inability to stand up/walk; speech difficulty and dizziness, as well as how fast a potential headache has developed, its location and its severity, might increase the proportion of neurologically intact patients that are recognized early. This in turn could potentially lead to a faster neurosurgical admission and improved outcome. A different approach would be to incorporate artificial intelligence. This has successfully been done to identify cardiac arrest during the emergency calls. Here, supporting the EMD with a machine learning system had a higher sensitivity and shorter time to recognition than EMDs alone [25, 26].

## Conclusion

Headache, nausea/vomiting and neck pain had the highest sensitivities and strongest associations with SAH in emergency medical calls. Headache and nausea/vomiting in combination was reported by more than 40%. Ninety percent of headaches were severe. Unspecific symptoms such as sweating, speech difficulty or inability to stand up or walk were each reported in 1 out of 5 calls and were also associated with SAH. Interestingly, 29.7% of conscious patients did not report headache as a symptom. A broad spectrum of symptoms makes early recognition of SAH a major challenge during emergency calls.

## Data Availability

The data that support the findings of this study are available from the national registries, Copenhagen Emergency Medical Services and medical records, but restrictions apply to the availability of these data. Data can be accessed with the necessary permits, and so are not publicly available. The authors are not authorized to pass on data without these permits.

## Abbreviations

SAH: Spontaneous subarachnoid haemorrhage
SE: Sensitivity
OR: Odds ratio
EMS: Emergency medical service
EMD: Emergency medical dispatch
EMDC: Emergency medical dispatch center
ICD-10: International Classification of Diseases version 10
LE: Laurits Elgaard (author)
SE: Sarita Egholm (author)
RedCap: Research Electronic Datacaptur Datacapture v. 10.3.3 by Vanderbilt University
AS: Asger Sonne (author)
VE: Vagn Eskesen (author)
AJ: Alice Jensen (author)
NB: Niklas Breindahl (author)
κ: Kappa
χ^2^: Chi-square
*P*: *P* Value
CI: Confidence interval
n: Number
