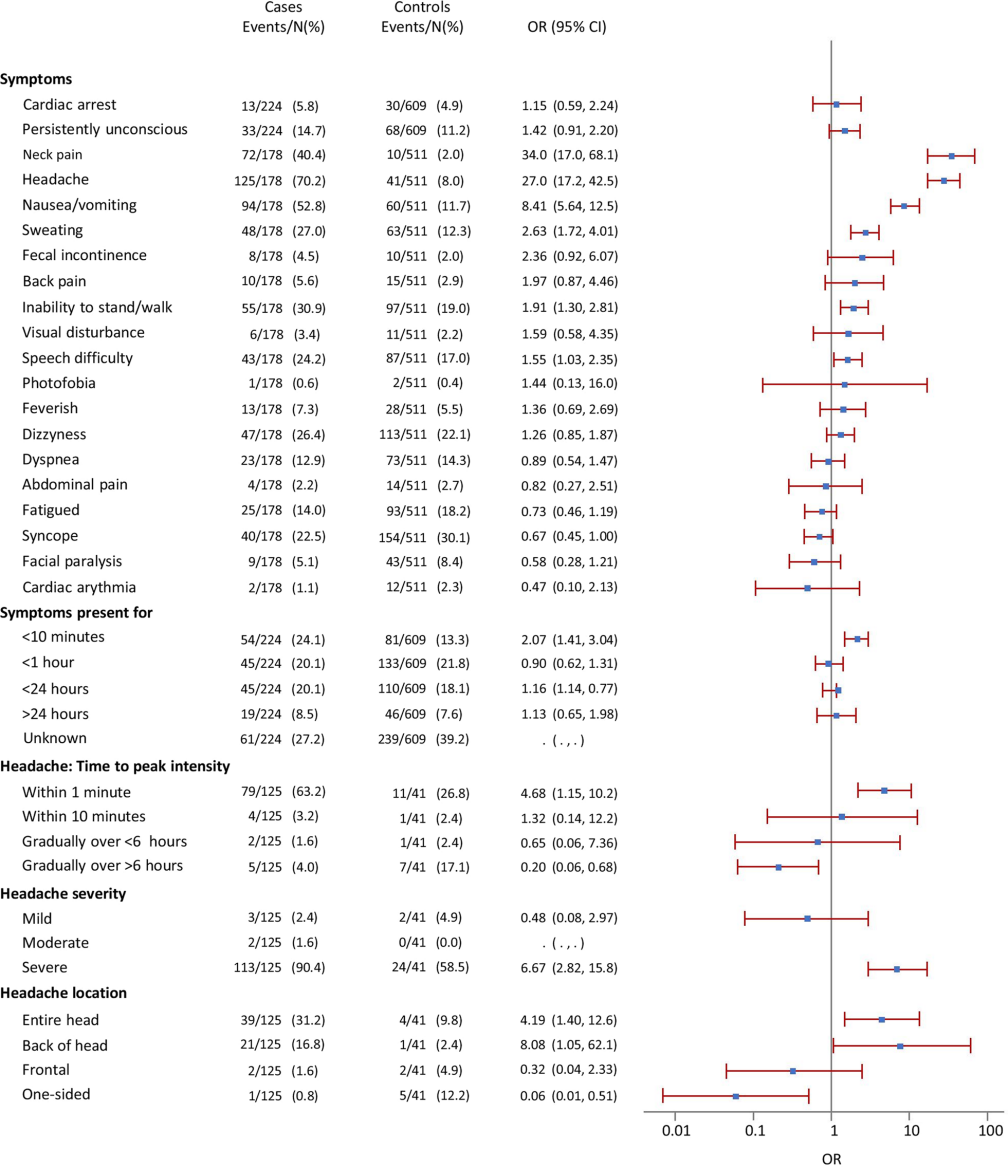# Correction to: Symptoms presented during emergency telephone calls for patients with spontaneous subarachnoid haemorrhage

**DOI:** 10.1186/s13049-021-00962-7

**Published:** 2021-10-14

**Authors:** Asger Sonne, Sarita Egholm, Laurits Elgaard, Niklas Breindahl, Alice Herrlin Jensen, Vagn Eskesen, Freddy Lippert, Frans Boch Waldorf, Nicolai Lohse, Lars Simon Rasmussen

**Affiliations:** 1 Department of Anaesthesia, Section 6011, Center of Head and Orthopaedics, Rigshospitalet, Inge Lehmanns Vej 6, 2100 Copenhagen, Denmark; 2 Department of Neurosurgery, The Neuroscience Centre, Rigshospitalet, University of Copenhagen, Copenhagen, Denmark; 3 Copenhagen Emergency Medical Services, Copenhagen, Denmark; 4 Research Unit of General Practice, Department of Public Health, University of Southern Denmark, Odense, Denmark; 5 The Research Unit for General Practice and Section of General Practice, Department of Public Health, University of Copenhagen, Copenhagen, Denmark; 6 Department of Emergency Medicine, Copenhagen University Hospital – Nordsjællands Hospital, Hillerød, Denmark; 7 Department of Clinical Medicine, University of Copenhagen, Copenhagen, Denmark

## Correction to: Scand J Trauma Resusc Emerg Med (2021) 29:118 https://doi.org/10.1186/s13049-021-00934-x

Following the publication of the original article [1], the authors informed as of an error in Figure 2: The term “persistently unconscious” had accidentally replaced “neck pain” in the third row under “Symptoms”.

The correct figure is shown here below and has already been included in the original article.